# Familial cardiac myxoma with multifocal recurrences: a case report and review of the literature

**DOI:** 10.1016/S1674-8301(11)60049-3

**Published:** 2011-09

**Authors:** Hailong Cao, Yanhu Wu, Jinfu Zhu, Yijiang Chen

**Affiliations:** a Department of Thoracic and Cardiovascular Surgery, the Affiliated Drum Tower Hospital of Nanjing University Medical School, Nanjing, Jiangsu 210008, China;; b Department of Thoracic and Cardiovascular Surgery, the First Affiliated Hospital with Nanjing Medical University, Nanjing, Jiangsu 210029, China

**Keywords:** cardiac myxoma, familial, multifocal, recurrence

## Abstract

We report a case of myxoma with multiple recurrences in both the atrium and ventricle in a 26-year-old woman five years after the surgical removal of left atrial myxoma. Her 52-year-old mother had a similar medical history. To our knowledge, this was the first familial case who suffered multifocal cardiac myxoma recurrences without any sign of the myxoma complex. Based on our understanding of the mechanism of recurrence, the approaches to prevent the recurrence, and markers to predict recurrence, we propose that multifocal recurrences, as reported herein, may result from a combination of familial predisposition and multifocal onset. The bi-atrial surgical approach and transesophageal echocardiography are preferred for patients with recurrent cardiac myxomas, especially for those with multiple recurrences and familial myxoma. Immunological and genetic screenings may help to identify family members at risk for developing this disease.

## INTRODUCTION

Cardiac myxoma is the most frequent primary cardiac neoplasm, with an estimated incidence of 0.5 per million populations per year, and usually with benign behavior[1]. Complete surgical removal of the tumor and its cardiac attachment is usually curative. However, reports of occasional postoperative recurrence indicate that it can sometimes develop a more aggressive course[2]. Myxoma is commonly classified into simple (or sporadic) cardiac myxoma and complicated cardiac myxoma. The latter includes myxoma complex, familial myxoma and myxoma from multi-centers, and represents about 7% of all cardiac myxomas[1]. Sporadic cardiac myxoma is usually found in middle-aged women, most in the left atrium (LA) as a solitary tumor, at a recurrence rate of 1%-3%. The complicated cardiac myxoma can be found in all ages and especially young women. It is often multi-centric at a recurrence rate of 10%-21%[3]. In this report, we describe a rare case of familial cardiac myxoma who suffered multifocal recurrences without any clinical sign of myxoma complex.

## CASE REPORT

**Fig. 1:**
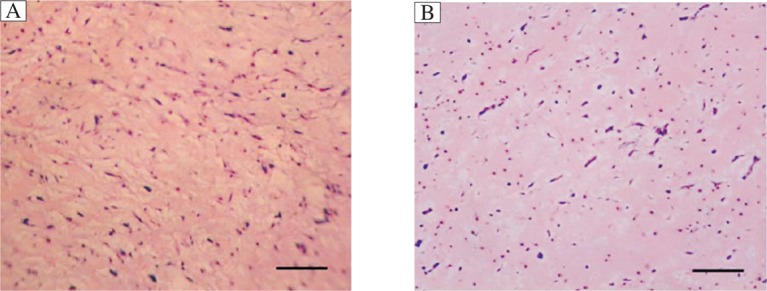
Representative histology of the cardiac myxoma tissue in a 26-year old woman with multiple recurrences of cardiac myxoma (HE, ×100). A: tumor in 2005. B: tumor in 2010. A similar histological appearance with irregular and papillary proliferations in the myxoid stroma between A and B. Bar = 30 µm.

A 26-year-old woman presented with exertional dyspnea, palpitation and fatigability in February 2010. Trans-thoracic echocardiography (TTE) at the First Hospital of Huaian indicated a recurrent myxoma in the left atrium. She was then referred to the First Affiliated Hospital of Nanjing Medical University, Nanjing, China, on March 3, 2010. The family history revealed that her 52-year-old mother had a history of left atrium myxoma and received cardiac surgery 12 years ago. Both the patient and her mother had similar symptoms, but the patient was more seriously ill about 5 years ago. At that time, TTE revealed a mass in the left atrium, which was attached to the inferior septum near the anterior mitral valve (MV) leaflet by a narrow stalk. The 12-lead ECG recording indicated that she had sinus tachycardia, right ventricular hypertrophy and the depression of the ST segments in lead II, III, a VF and V4- V6. Cardiac surgery was performed in 2005 through a trans-septal approach with the use of extracorporeal circulation. A left atrium myxoma (40 mm×30 mm×30 mm) attached to the inferior septum near the mitral annulus was excised with the adjacent normal tissue, and the resultant defect was closed with a running suture. Pathological analysis confirmed the diagnosis of myxoma (***Fig. 1A***). The patient was discharged 20 d after the operation. TTE in 2007 revealed no recurrence.

Five years after her first surgery, physical examination showed normal jugular venous pressure. Auscultation revealed an early moderate diastolic murmur (“plop”) at the apex, which changed with body posture. The patient or her first-degree relatives did not have lentiginosis, cutaneous myxomas, or any endocrine abnormalities. Laboratory examination showed mild anemia with increased erythrocyte sedimentation rate (ESR, 36 mm/h), hemoglobin (10.5 mg/dL), and hematocrit (32%). ECG showed normal sinus rhythm, normal axis and no significant pathological change. Chest X-ray was also within normal limits.

TTE confirmed a pediculated mass (41 mm×29 mm) in the left atrium, which was attached to the septum. The tumor prolapsed from the left atrium into the left ventricle through the mitral valve orifice in early diastole. A CDFI and PDE mode echocardiograph also showed mild mitral and tricuspid regurgitation. At d 8, surgical excision of the intracardiac myxoma was performed through median sternotomy accompanied by moderate hypothermia and cardiopulmonary bypass. An incision was made in the right atrium and a trans-septal approach was also used. A big jelly-like left atrial mass (40 mm×35 mm) was seen with the attachment to the interatrial septum in the same location as the previous lesion. An additional myxoma (4.0 mm×4.0 mm) was found in the upper lateral wall of the left atrium chamber. The small myxoma with the surrounding normal tissue was completely excised. The big myxoma was removed with approximately 1 cm of the atrial tissue at the base. After excision of the recurrent myxomas, copious irrigation of the atria and ventricles with saline solution was performed to eliminate any tumor fragments that might have been dislodged during removal of the tumor. After the cardiac chambers were inspected and no additional tumors were found, the atrial septal defect was closed with a Teflon patch. The duration of cardiopulmonary bypass was 70 min and clamp time was 38 min. The duration of ventilation was 13 h and intensive care unit stay was 16 h. Five units of red blood cells, 600 mL plasma and 10 units of platelet were infused.

On pathological examination, the main specimen consisted of a partially hemorrhagic, soft, yellow mass measuring 40 mm×30 mm×30 mm, and the small one was a diaphanous and gelatinous tissue. Microscopically, both tumors showed similar histological appearance with irregular and papillary proliferations in the myxoid stroma, and were diagnosed as benign myxoma. There was no evidence of residual tumor in the surgical margins. No mitosis or pleomorphism was observed (***Fig. 1B***).

The postoperative course was uneventful, but a routine TTE prior to discharge found another mass (9.0 mm×9.7 mm) at the junction between the free margin of the posterior mitral valve leaflet and chordae tendineae; a blind field during surgery through a trans-septal approach. It was further confirmed by trans-esophageal echocardiography (TEE). We recommended additional surgery for the patient to remove the myxoma, but it was rejected by the patient because of her severe emotional stress.

## DISCUSSION

In 1954, Clarence Crafoord, the pioneer Swedish cardiovascular surgeon, removed an intra-atrial myxoma for the first time. Follow-up 38 years later found the patient alive and well[4]. Since then, it has been generally believed that myxomas never recur. Based on the long-term follow-up and pathological behavior of myxoma, Firor *et al*.[5] and Malm *et al*.[6] proposed that simple excision of atrial myxomas was adequate and resection of the adjacent atrial septum or wall was unnecessary. However, cases of recurrence of myxoma have been occasionally reported. For instance, Gerbode and colleagues[7] reported a case of recurrent atrial myxoma 4 y postoperatively. Kabbani *et al*.[8] reported a young woman with multiple recurrent left atrium myxomas 1.5 y postoperatively.

Based on our search in both English and Chinese literature, we summarize all the case reports, including the present case, in ***Table 1***. Of the 21 patients with multiple recurrent myxomas, the age range was between 16 and 58 y and the female/male ratio was 16/21 (76% females). The time interval between the first intervention and the recurrence was from 3 mon to 14.5 y with an average of 4.2 y. Of all the cases in ***Table 1***, the primary tumors were 71% (15/21) solitary in the left atrium and 10% (2/21) in the right atrium, and 19% (4/21) multifocal in the left atrium and/or other cardiac chambers. The recurrent tumors appeared in the left atrium or both atria in 9 (43%) patients and in the left and/or right ventricle in 12 (57%) patients. Among them, 7 (33%) patients experienced a second recurrence. Seven (33%) patients had a familial history of cardiac myxoma, and 4 of them were diagnosed as Carney complex (major manifestations including cardiac myxoma, non-cardiac myxomas, spotty pigmentation, endocrine abnormalities and neural disease)[9]. Our case is the only familial case who suffered multifocal cardiac myxoma recurrences without any evidence of the myxoma complex.

**Table 1:** Reported cases of multiple recurrent myxomas

Number	Reference	Age (y)	Gender	Familial myxoma	Site of primary myxoma	Recurrence time	Site of recurrence
1	Kabbani *et al.* (1973) [8]	19	F	-	LA	1 y 6 mon	LA multiple
2	Jugdutt *et al.* (1975) [19]	46	M	-	LA	3 y 5 mon	LA multiple, RA*
3	Sasaki *et al.* (1977) [20]	32	M	-	LA	8 mon	LA multiple
4	Grauer *et al.* (1983) [21]	16	F	-	LA	1 y 5 mon	LA, RA
5	Hada *et al.* (1984) [22]	23	F	-	LA	4 y	LA, RA, RV
6	Gray *et al.* (1985) [23]	32	M	-	LA	3y 9mon	LA, RA, RV, PA*
7	Ohshima *et al.* (1990) [24]	27	F	Familial	LA	4 y	LA, RA, RV
8	Selke *et al.* (1990) [3]	30	F	-	RA	4 y	RA, LA*
9	Goldstein *et al.* (1995) [25]	47	F	-	LA, MV	3 mon	LA, MV, LPV
10	Aroca *et al.* (1996) [26]	58	F	-	LA	5 y	LA, RA
11	Saurbier *et al.* (1997) [27]	36	F	Familial	LA	9 y	LA multiple, RA
12	Reber *et al.* (2001) [2]	15	F	-	RA	2 y	RA multiple
13	Hermans *et al.* (2003) [28]	58	M	-	LA	14 y 6 mon	LV multiple*
14	Akbarzadeh *et al.* (2005) [29]	35	F	Carney	LA	2 y	LA multiple, LV*
15	Kojima *et al.* (2005) [17]	43	F	Carney	LA	3 y 4 mon	LA, LV multiple
16	Moyssakis *et al.* (2005) [30]	21	M	-	RA, RV	11 y	LA, RA*
17	Ito *et al.* (2006) [31]	26	F	Carney	LA	4 y	LA multiple, RV
18	Vargas *et al.* (2008) [9]	18	F	-	LA, RA	3 y	LA, LV, RV
19	Vargas *et al.* (2008) [9]	36	F	Carney	LA, RA, LV, RV	2 y	LA, RA, RV
20	Tang *et al.* (2010) [32]	33	F	-	LA	4 y	RA, RV multiple*
21	Present case (2010)	26	F	Familial	LA	4 y 11 mon	LA multiple, LV

*Cases with a second recurrence. MV: mitral valve; LA: left atrium; LV: left ventricle; RA: right atrium; RV: right ventricle; LPV: left pulmonary vein; PA: pulmonary artery; F: female; M: male.

The mechanism of recurrence is still not clear. The following factors should be considered. First, the original tumor may re-grow due to incomplete resection. Second, there may be a familial predisposition for recurrence. Third, embolic fragments of the original tumor may be implanted in the myocardium spontaneously or due to a previous surgery. Forth, a pretumorous focus may be present in another part of the myocardium. In the present case, the location of the main recurrent myxoma in the left atrium was just the same as that of the primary one, possibly indicating an incomplete resection. However, this cannot explain the other two myxomas in the left atrium and the left ventricle. Therefore, it is most likely due to familial predisposition and multi-centre onset. Another possible explanation of the multiple recurrences proposed recently is malignant transformation. Mendoza and coworkers[10] reported that the first and second recurrent cardiac myxomas had a more aggressive histologic appearance in a second multiple recurrent case. However, the present case showed the same histological appearances as the primary myxoma without any sign of malignancy (***Fig. 1***).

To reduce the risk of recurrence, we recommend total extirpation of the tumor with the base in full thickness and with resection of the healthy tissue around the tumor (at least 5 mm) and copious irrigation of the cardiac chambers. In addition, the surgical approach chosen for resecting cardiac tumors should have clean surgical margins and the cardiac chambers should be carefully explored to preclude multiple heterotopic tumors. Bi-atrial approach allows the inspection of the four cardiac chambers, limits manipulation of the mass, and facilitates the complete excision of the tumor. Thus, this should be used when a patient has myxomas in multiple cardiac chambers or exhibits a definite familial nature[11]. Alternatively, heart transplantation can be considered as an alternative therapy to prevent recurrence when the primary or recurrent myxoma is too difficult to be removed[12].

Echocardiography is currently the most important diagnostic tool for imaging cardiac myxomas. It is accurate, dependable, and noninvasive, and does not entail any risk of tumor fragmentation and embolization. Currently, the decision for surgery is generally based on echocardiographic evidence of the disease. In a retrospective multi-center study, diagnosis of cardiac myxomas has been achieved by TTE in 95.2%, and TEE in 100%[13]. In fact, TEE is superior to TTE in many ways. First, TTE may miss a tumor less than 5 mm in diameter and sometimes even larger tumors. Second, TEE provides greater anatomic detail regarding the size and site of attachment, and exclusion of lesions elsewhere[14]. Third, in the case of valvular myxoma, TEE can guide the surgical approach by revealing the integrity and mobility of the valve prior to operation, and assess repair of the valve during surgery[15]. Indeed, if TEE was performed before operation or during surgery in the present case, the myxoma in the left ventricle would be detected and resected simultaneously with the other two left atrial myxomas.

Whether there is a marker for predicting the occurrence and recurrence of cardiac myxomas is still an open question. Keeling and coworkers[16] found significant immunological alterations in myxoma patients that seemed to occur pre- and postoperatively, including serum protein electrophoresis, C-reactive protein, fluorescence-activated cell sorter, interleukin 2 receptor, and intracellular adhesion molecule (ICAM). In cases of recurrence, these parameters may be altered again. In addition, they also found alteration of ICAM in up to 66.6% of long-term survivors after resection of a cardiac myxoma without recurrence of the disease[16]. Recently, genetic difference between patients with familial and non-familial cardiac myxomas has been identified. A novel frame shift mutation in exon 2 of protein kinase A regulatory subunit 1α (*PRKAR1A*) in a heterozygous fashion was detected to be associated with genesis of familial cardiac myxoma (especially Carney complex)[17], and *PRKAR1A* haploinsufficiency was found to be associated with recurrence of cardiac myxomas[18]. Therefore, immunological and genetic screening of patients with recurrent cardiac myxomas may help identify patients at risk for additional recurrence.

In conclusion, multifocal cardiac myxomas seem more often in young women, in patients with multifocal origins, and in those who have a family history of the tumor. The biatrial surgical approach and TEE are recommended for patients with recurrent cardiac myxomas, especially for multiple recurrences and familial myxoma. Additional immunological and genetic screening may help to identify family members at risk for the disease and recurrence.
